# Supplementary material for: Evidence supported by Mendelian randomization: impact on inflammatory factors in knee osteoarthritis
Source: Front Med (Lausanne). 2024 May 28;11:1382836. doi: 10.3389/fmed.2024.1382836 (PMC11165061; doi:10.3389/fmed.2024.1382836)
Supplement: Supplementary file 1 [file Data_Sheet_1.ZIP › data/GCST90274782_5e6/782.leaveoneout.pdf]

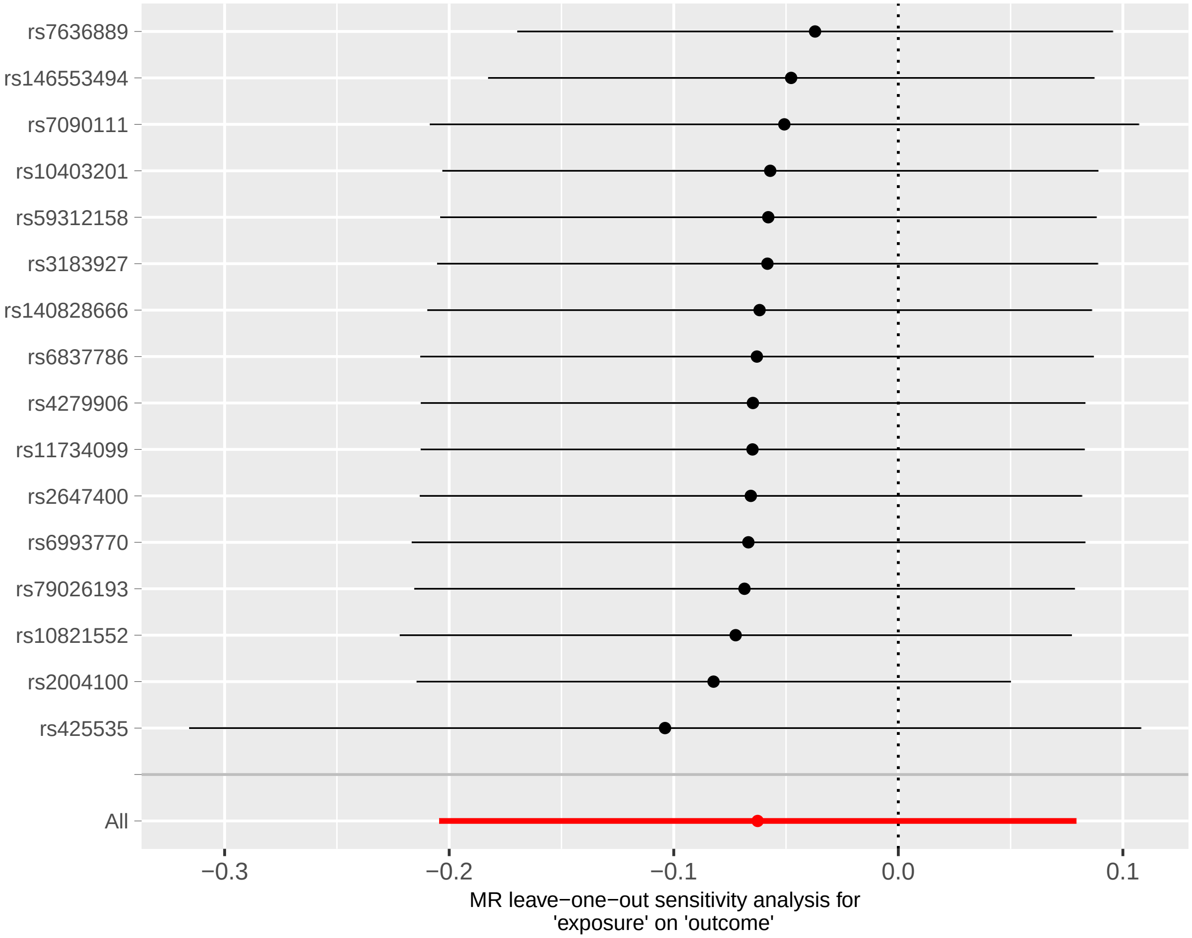

rs7636889
rs146553494
rs7090111
rs10403201
rs59312158
rs3183927
rs140828666
rs6837786
rs4279906
rs11734099
rs2647400
rs6993770
rs79026193
rs10821552
rs2004100
rs425535
All
−0.3
−0.2
−0.1
0.0
0.1
MR leave−one−out sensitivity analysis for
'exposure' on 'outcome'
